# P-2205. Factors Associated with Human Herpesvirus 6 DNAemia Among Allogeneic Hematopoietic Cell Transplant Recipients in the Modern Era

**DOI:** 10.1093/ofid/ofaf695.2368

**Published:** 2026-01-11

**Authors:** Yusuke Ohashi, Roy F Chemaly, Fareed Khawaja, Marilyne Daher, Amy Spallone, Tali Shafat, Ella Ariza Heredia, Guy Handley

**Affiliations:** The University of Texas MD Anderson Cancer Center, Houston, TX; The University of Texas MD Anderson Cancer Center, Houston, TX; The University of Texas MD Anderson Cancer Center, Houston, TX; Infectious Diseases, Infection Control, and Employee Health, Houston, Texas; University of Texas MD Anderson Cancer Center, Houston, Texas; The University of Texas MD Anderson Cancer Center, Houston, TX; The University of Texas MD Anderson Cancer Center, Houston, TX; The University of Texas MD Anderson Cancer Center, Houston, TX

## Abstract

**Background:**

Human herpesvirus 6 (HHV-6) DNAemia frequently occurs following allogeneic hematopoietic cell transplantation (allo-HCT). Prior studies have established risk factors for DNAemia. However, advances such as haploidentical HCT, post-transplant cyclophosphamide (PtCy) for graft-versus-host disease (GVHD) prevention, and CMV prevention may have affected the risk of DNAemia.

**Table 1.**
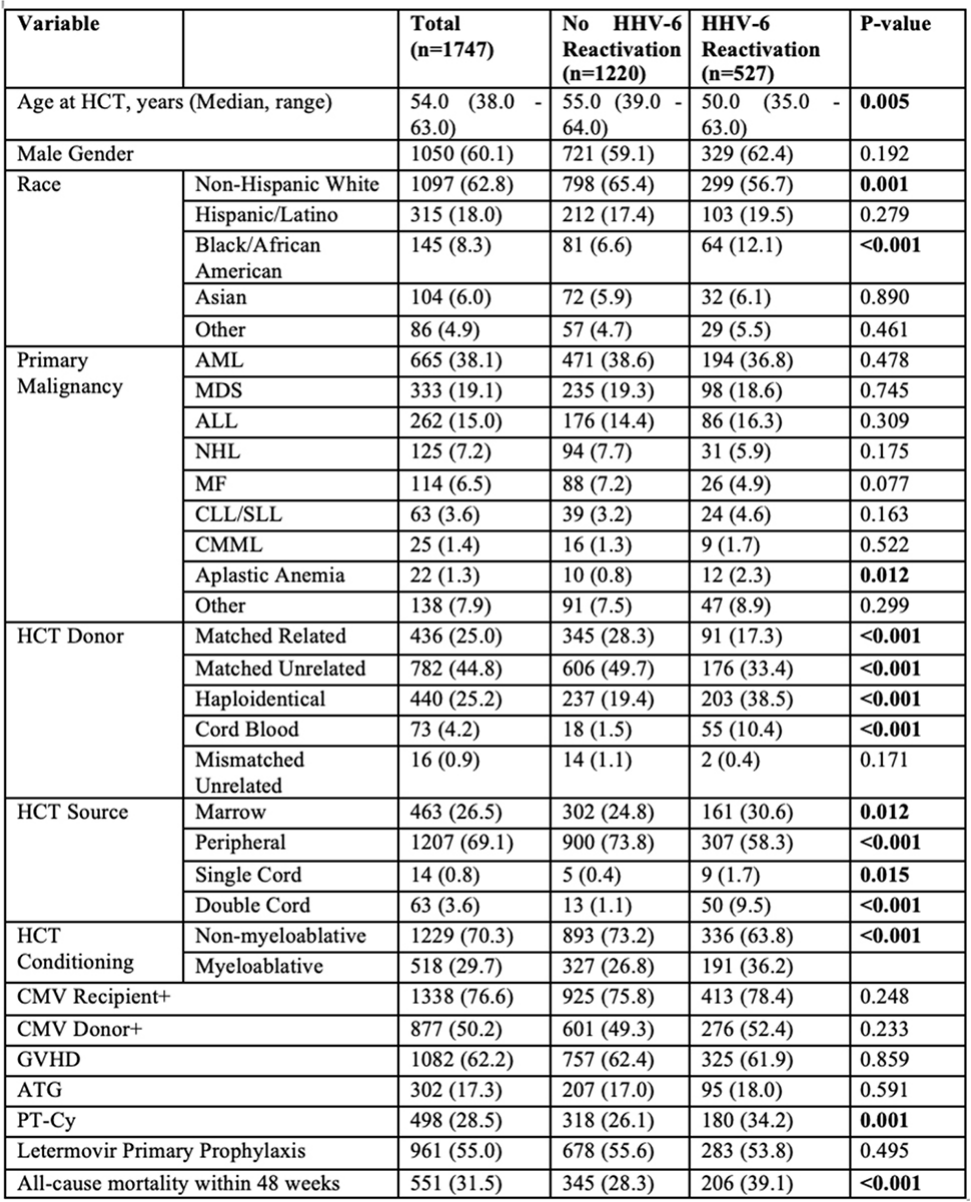
Baseline characteristics of allogeneic hematopoietic cell transplant recipients. Abbreviations: ALL, acute lymphocytic leukemia; AML, acute myeloid leukemia; ATG, antithymocyte globulin; CDV, cidofovir; CLL, chronic lymphocytic leukemia; CML, chronic myeloid leukemia; CMML, chronic myelomonocytic leukemia; CMV, cytomegalovirus; DNA, deoxyribonucleic acid; EBV, Epstein-Barr virus; FOS, foscarnet; GCV, ganciclovir; GVHD, graft-versus-host disease; HCT, hematopoietic stem cell transplantation; MDS, myelodysplastic syndrome; MMUD, mismatched unrelated donor; MRD, matched related donor; MUD, matched unrelated donor; NHL, non-Hodgkin lymphoma; PCR, polymerase chain reaction, PT-Cy, post-transplant cyclophosphamide; SD, standard deviation; SLL, small lymphocytic lymphoma; VGCV, valganciclovir.

**Table 2.**
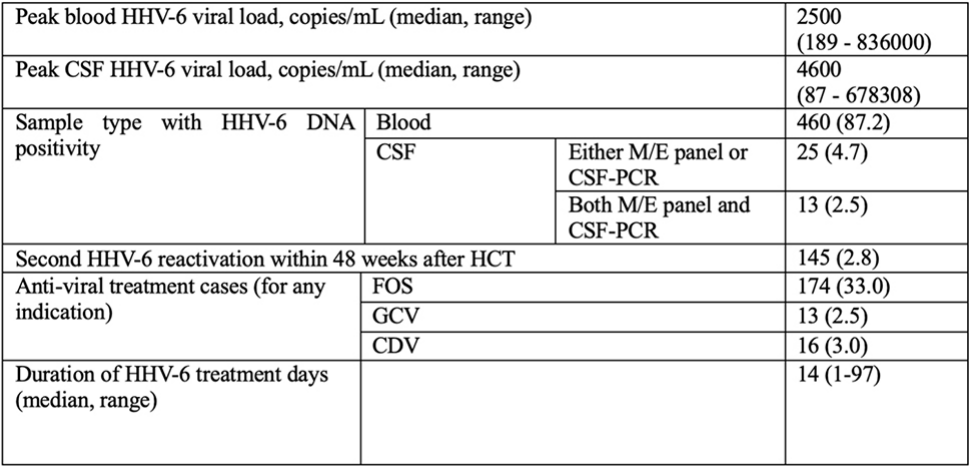
Characteristics of HHV-6 reactivation (n=527) Abbreviations: CDF, cidofovir; CSF, cerebrospinal fluid; FOS, foscarnet; GCV, ganciclovir; M/E: Meningitis/encephalitis VGCV, valganciclovir.

**Methods:**

In this retrospective single-center cohort study, patients who underwent allo-HCT from 03/2016 to 02/2023 with at least one assessment for HHV-6 DNA by PCR plasma testing within 48 weeks post-transplant. Demographic, clinical and laboratory data were evaluated to determine associations with DNAemia.

**Figure:**
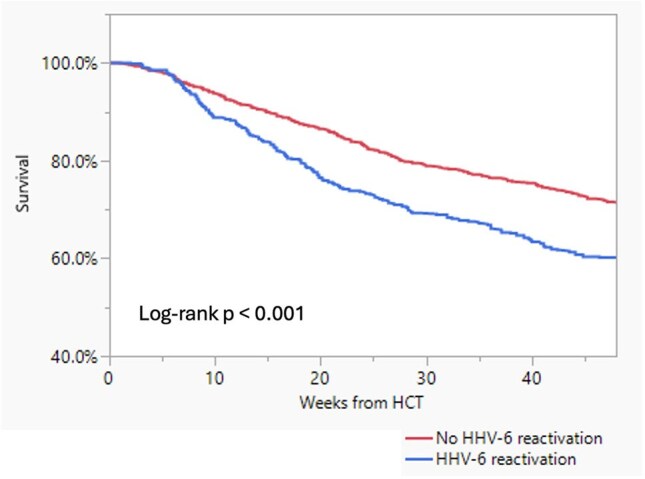
Rate of survival from HCT over 48 weeks in patients with and without HHV-6 reactivation

**Results:**

Antiviral therapy was initiated for 188 (35.7%) patients with DNAemia, mostly with foscarnet (174/188). ACM within 48 weeks of transplantation was higher in patients with DNAemia (39.1% vs 28.3%; p< 0.001), and in those patients with DNAemia who received therapy (50.0% vs 33.0%; p< 0.001).

**Conclusion:**

The prevalence of HHV-6 DNAemia was high in our cohort but with low incidence of encephalitis. Although, all cause mortality was higher in patients with HHV-6 DNAemia, patients who received therapy for DNAemia had worse outcomes. While receipt of post-transplant cyclophosphamide was associated with increased DNAemia, no association was found with ATG use and primary letermovir prophylaxis.

**Disclosures:**

Roy F. Chemaly, MD, MPH, FIDSA, FACP, FESCMID, ADMA Biologics: Advisor/Consultant|AiCuris: Advisor/Consultant|AiCuris: Grant/Research Support|Ansun Biopharma: Advisor/Consultant|Ansun Biopharma: Grant/Research Support|Assembly Bio: Advisor/Consultant|Astellas: Advisor/Consultant|Eurofins Viracor: Advisor/Consultant|Eurofins Viracor: Grant/Research Support|Genentech: Grant/Research Support|Gilead: Advisor/Consultant|InflaRX: Advisor/Consultant|IntegerBio: Advisor/Consultant|Karius: Advisor/Consultant|Karius: Grant/Research Support|Merck/MSD: Advisor/Consultant|Merck/MSD: Grant/Research Support|Moderna: Advisor/Consultant|Oxford Immunotec: Advisor/Consultant|Pfizer: Advisor/Consultant|Shionogi: Advisor/Consultant|Takeda: Advisor/Consultant|Takeda: Grant/Research Support|Tether: Advisor/Consultant Fareed Khawaja, MBBS, Eurofins Viracor: Grant/Research Support|MERCK: Grant/Research Support|Symbio: Grant/Research Support

